# Successful isolation of *Treponema pallidum* strains from patients’ cryopreserved ulcer exudate using the rabbit model

**DOI:** 10.1371/journal.pone.0227769

**Published:** 2020-01-13

**Authors:** Lara E. Pereira, Samantha S. Katz, Yongcheng Sun, Patrick Mills, Willie Taylor, Patricia Atkins, Charles M. Thurlow, Kai-Hua Chi, Damien Danavall, Nicholas Cook, Tamanna Ahmed, Alyssa Debra, Susan Philip, Stephanie Cohen, Kimberly A. Workowski, Ellen Kersh, Yetunde Fakile, Cheng Y. Chen, Allan Pillay

**Affiliations:** 1 Division of STD Prevention, Centers for Disease Control and Prevention, Atlanta, GA, United States of America; 2 Division of Scientific Resources, Centers for Disease Control and Prevention, Atlanta, GA, United States of America; 3 Charles River Laboratories, Wilmington, MA, United States of America; 4 Oak Ridge Institute for Science and Education, Oak Ridge, TN, United States of America; 5 San Francisco Department of Public Health, San Francisco, CA, United States of America; 6 Emory University Department of Medicine, Atlanta, GA, United States of America; University of Lincoln, UNITED KINGDOM

## Abstract

Clinical isolates of *Treponema pallidum* subspecies *pallidum* (*T*. *pallidum*) would facilitate study of prevalent strains. We describe the first successful rabbit propagation of *T*. *pallidum* from cryopreserved ulcer specimens. Fresh ulcer exudates were collected and cryopreserved with consent from syphilis-diagnosed patients (N = 8). Each of eight age-matched adult male rabbits were later inoculated with a thawed specimen, with two rabbits receiving 1.3 ml intratesticularly (IT), and six receiving 0.6 ml intravenously (IV) and IT. Monitoring of serology, blood PCR and orchitis showed that *T*. *pallidum* grew in 2/8 rabbits that were inoculated IV and IT with either a penile primary lesion specimen (CDC-SF003) or a perianal secondary lesion specimen (CDC-SF007). Rabbit CDC-SF003 was seroreactive by *T*. *pallidum* Particle Agglutination (TP-PA) and Rapid Plasma Reagin (RPR) testing, PCR+, and showed orchitis by week 6. Euthanasia was performed in week 7, with treponemal growth in the testes confirmed and quantified by qPCR and darkfield microscopy (DF). Serial passage of the extract in a second age-matched rabbit also yielded treponemes. Similarly, rabbit CDC-SF007 showed negligible orchitis, but was seroreactive and PCR+ by week 4 and euthanized in week 6 to yield *T*. *pallidum*, which was further propagated by second passage. Using the 4-component molecular typing system for syphilis, 3 propagated strains (CDC-SF003, CDC-SF007, CDC-SF008) were typed as 14*d*9f, 14*d*9g, and 14*d*10c, respectively. All 3 isolates including strain CDC-SF011, which was not successfully propagated, had the A2058G mutation associated with azithromycin resistance. Our results show that immediate cryopreservation of syphilitic ulcer exudate can maintain *T*. *pallidum* viability for rabbit propagation.

## Introduction

For the past several decades, the rabbit has been the primary animal model for the study of syphilis pathogenesis and its causative agent, *Treponema pallidum* subspecies *pallidum* (hereafter referred to as *T*. *pallidum*). Research on the utility of various culture media and methods for *in vitro* propagation have yielded inconsistent results over the years. Sustained passage has been limited or unattainable, with low yields, contamination, and/or loss of viability, virulence and pathogenicity being reported [1–7]. However, a recent study suggests that *in vitro* propagation of *T*. *pallidum* is possible using a microaerobic, nutrient-defined rabbit cell culture system, with sustained propagation of viable treponemes for >6 months [8]. Further refinement of the culture system is ongoing to determine its applicability for propagation of *T*. *pallidum* strains directly from clinical specimens. In the interim, the *in vivo* rabbit model remains the standard method for propagating viable treponemes and/or testing treponemal infectivity via intra-testicular (IT), intravenous (IV), intradermal or intracisternal inoculation routes [9]. Orchitis, serology and/or polymerase chain reaction (PCR) of blood specimens are performed to provide qualitative and quantitative measures of infection. Similar to humans, treponemal and non-treponemal antibody responses develop in rabbits following *T*. *pallidum* infection and can be detected using many of the same serology assays used for patient screening that include Rapid Plasma Reagin (RPR) and *Treponema pallidum* particle agglutination (TP-PA) [10–12].

The study of *T*. *pallidum* is particularly relevant today, given the rise in syphilis rates among both men and women and most age groups across the United States, as well as the emergence of ocular syphilis clusters in recent years [13–15]. Indeed, the syphilis rabbit model has provided much insight on ocular and neuroinvasive *T*. *pallidum* strains and their clinical manifestations, and has also facilitated study of syphilis vaccines and the efficacy and/or resistance profiles of antibiotics used for syphilis treatment [16–26]. Research, diagnostics and surveillance studies would benefit from a sustained supply of *T*. *pallidum* stocks, as sufficient biological material is necessary for assay development, whether as a source of antigen(s) for serology tests, or gene targets for molecular assays. Indeed, molecular amplification techniques have facilitated the study of specific *T*. *pallidum* genes linked to antibiotic resistance, virulence, and pathogenicity [5, 21, 27–34]. Species subtyping in particular differentiates among strains of *T*. *pallidum* which is important for syphilis epidemiological investigations, where information about related or new emerging strains may prove useful for surveillance, diagnosis, prevention and treatment [5, 35–37]. The availability of the complete sequence of the *T*. *pallidum* genome and the advent of automated whole genome sequencing (WGS) [5, 28, 37–46] have created opportunities to develop new and/or improved molecular genetic methods for syphilis diagnosis and genotyping, and requires clinical isolates of *T*. *pallidum* to further develop and refine these techniques. DNA Enrichment methods and phylogenomic analyses of *T*. *pallidum* from either direct patient specimens or strains propagated in rabbits have in recent years shed light on the evolutionary origins, antibiotic resistant profiles, and immune evasion mechanisms of circulating strains [31, 43, 47–49]. As some of these studies have shown, PCR analysis can be performed directly on DNA extracted from clinical specimens, but sample weights and/or volumes are often limited, hindering expanded testing, specimen archiving, and study of disease in animal models. Fresh specimens that include blood, cerebrospinal fluid (CSF), and lesion exudate from patients diagnosed with syphilis have been successfully passaged in rabbits to yield viable stocks of *T*. *pallidum* [9, 27, 31, 50] but the propagation of *T*. *pallidum* in the rabbit model is not without its challenges, as it is labor and time intensive with varying levels of success depending on strain, specimen quality, and conditions such as ambient temperature. Another major limiting factor is the need for proximity of animal research laboratories to clinics and hospitals for fresh patient specimen collection and inoculation into rabbits. A method to grow *T*. *pallidum* from frozen specimens would be ideal and is the objective of this study, as it would considerably widen the scope of syphilis research by eliminating the necessity for fresh specimens and proximity of animal laboratories to clinical specimen collection sites. We describe here the first successful rabbit propagation of *T*. *pallidum* from patients’ cryopreserved syphilitic ulcer specimens as part of a CDC advanced molecular detection (AMD) funded initiative.

## Materials and methods

### Patient cohort and specimen collection

Specimens utilized in this study were collected from the San Francisco Municipal STD Clinic in San Francisco, CA. At the clinic, which performs darkfield (DF) microscopy, 16 DF+ specimens were collected from January to August 2017. Each specimen was from a different patient. Project Determination Approval at CDC (PD# 6857) and local IRB approvals at San Francisco (IRB# 16–20056) were obtained prior to study initiation. Eligible subject populations included adults with a case of primary or secondary syphilis, presenting with exudative syphilitic lesions amenable to specimen collection. Participation was voluntary, required informed patient consent, and involved interpreters if requested by the patient. All patients received routine evaluation and care for syphilis regardless of study participation. During the physical examination, lesion exudate was collected from genital or anal ulcers, or condyloma lata that were DF+. For lesion exudate collection, the ulcer or lesion was first cleaned with a gauze pad moistened with sterile saline. The chancre was gently squeezed to release serous exudate while taking precautions to avoid blood contamination. A sterile Dacron swab was used to collect ulcer exudate by gently rolling the swab along the base of the ulcer and then immediately placing it into a cryovial containing storage medium warmed to room temperature after storage at -20°C. The storage medium consisted of 1 ml 50% v/v sterile glycerol and normal rabbit serum (NRS). The swab was then gently agitated in the cryovial and discarded. Cryovials containing exudate specimens were snap frozen in liquid nitrogen. All specimens were stored at -80°C until shipment on dry ice to the CDC. Specimens were stored in liquid nitrogen vapor phase at the CDC until use in rabbit model experiments.

### Rabbit experiments and specimen collection

**Fig 1:**
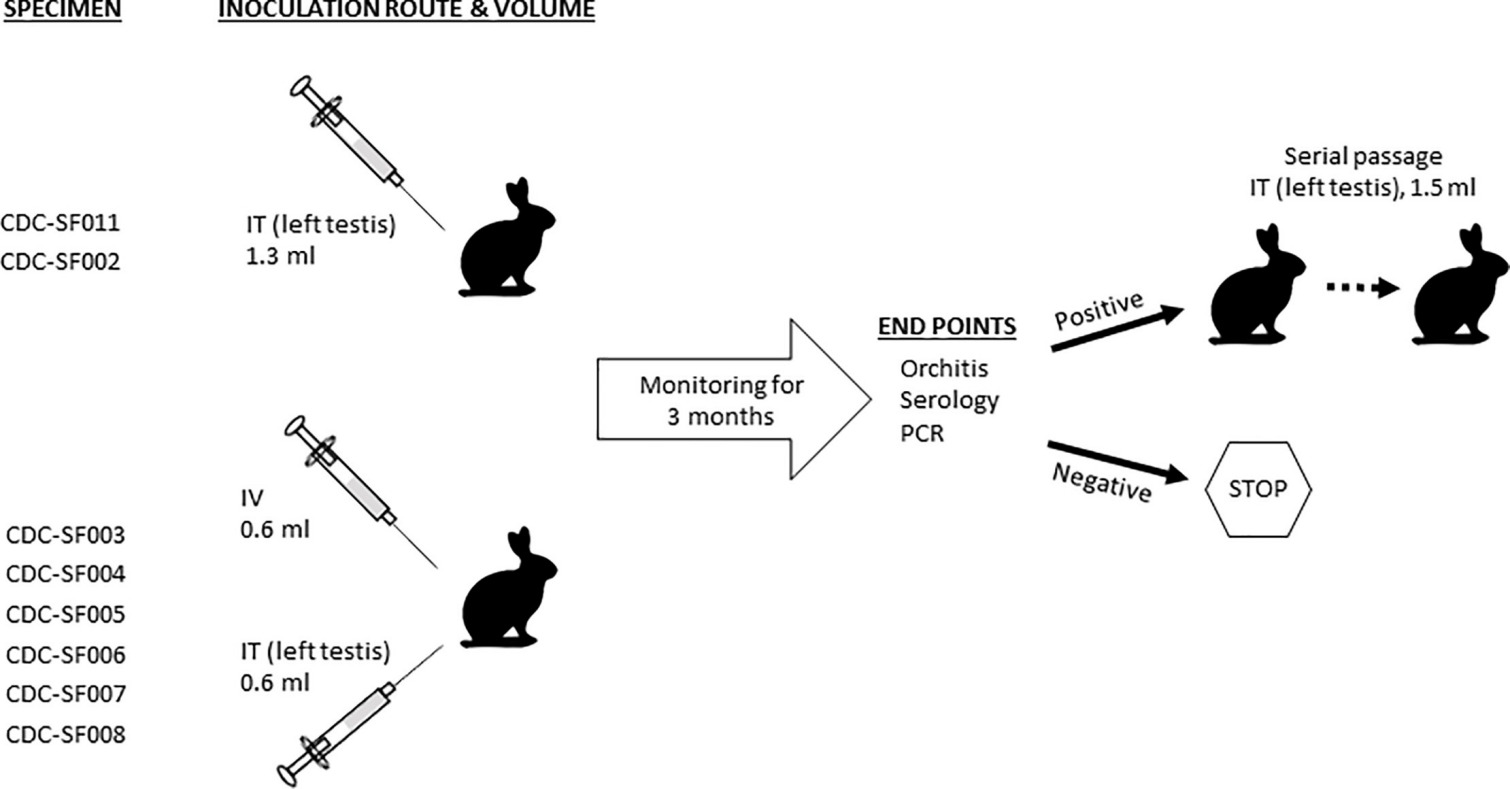
Schematic summarizing propagation of *T*. *pallidum* from patients’ lesion exudate specimens. Each of eight rabbits was inoculated with one of the specimens listed, and the injection was either administered IT in the left testis, or both IV and IT (left testis) at the volumes indicated. Each rabbit was then monitored once weekly for up to three months until results indicated a positive infection status by the parameters shown, at which time euthanasia was performed. If propagation occurred, up to two serial passage(s) were performed using fresh or frozen extract from the previous passage rabbit. Rabbits that remained negative by all three measures of infection were euthanized at the three-month mark with no further passage.

A total of 13 adult age-matched (8–9 months) male New Zealand White rabbits (*Oryctolagus cuniculus*) with an average body mass of 3 kg were utilized in this study (Fig 1). All rabbits were prescreened to confirm negative treponemal and nontreponemal antibodies status. Rabbits were housed under approved biosafety level 2 containment conditions at the CDC. Their diet, care, and maintenance conformed to the *Guide for the Care and Use of Laboratory Animals* guidelines [51]. All procedures outlined in this study were approved by the CDC Institutional Animal Care and Use Committee (IACUC Protocol #2979). Prior to procedures, animals were sedated with acepromazine (0.5–2 mg/kg body weight) intramuscularly. Eight patient specimens were selected for propagation (Fig 1, Table 1), with each rabbit being inoculated with a single specimen. Each inoculum was prepared by gently mixing 1 ml of frozen exudate specimen with 0.5 ml of pre-warmed (37°C) NRS. For two of the specimens, approximately 1.3 ml was injected in the left testicle of a rabbit, while for the remaining specimens, an equal volume of inoculum was injected intravenously (IV, 0.6ml) and intratesticularly (IT, 0.6ml, left testicle) as shown in Fig 1. The backs of the rabbits that were injected IV and IT were shaved to facilitate monitoring of disseminated skin lesions, if any, with clipping performed as needed to clear fur growth during the monitoring period [9]. Residual volume of each inoculum was retained for further analysis by quantitative PCR (qPCR) and DF microscopy at CDC. Blood was collected from each animal at baseline, once weekly thereafter, and at euthanasia for serology and PCR analysis using serum separation tubes (BD Biosciences, San Jose CA) and PAXgene blood DNA tubes (Qiagen, Germantown MD), respectively. Orchitis was monitored up to twice weekly. The animals were also monitored for the development of any non-syphilitic disease manifestation due to the potential presence of other infectious agents in the lesion exudates; none were observed over the course of the study. Endpoints for euthanasia were seroreactivity, development of orchitis, and/or positive blood PCR results. Rabbits were euthanized when these criteria were met, or at the end of 3 months if results remained negative; whichever occurred first [9]. Euthanasia (150 mg/kg beuthanasia IV) was performed according to AVMA Guidelines on Euthanasia [52]. *T*. *pallidum* was obtained from rabbit testes tissues and processed as previously described [9]. Testes tissues were immediately processed for PCR analysis and to extract treponemes for cryopreservation and further passage in rabbits. For rabbits that yielded viable *T*. *pallidum*, fresh or frozen extract (1.5 ml) from the first passage rabbit was serially propagated (IT, left testis) to generate more treponemes (Fig 1).

**Table 1:** Clinical and laboratory data for patient specimens used for *in vivo* rabbit propagation.

Specimen ID^1^	San Francisco Municipal STD Clinic				CDC Laboratory
	Syphilis stage	Site of lesion, ulcer/chancre/lesion	DF	Antibody titer^2^ (assay)	qPCR (genomic copies/ml)
CDC-SF011	Primary	Penile, sore	Positive	1:4 (RPR)	7.00 x 10^4^
CDC-SF002	Primary	Penile (glans), lesion	Positive	Weakly reactive (VDRL)	6.44 x 10^2^
CDC-SF003^3^	Primary	Penile (coronal sulcus), ulcer	Positive	1:4 (VDRL)	1.14 x 10^4^
CDC-SF004	Secondary	Scrotum, lesion	Positive	1:16 (VDRL)	7.58 x 10^4^
CDC-SF005	Primary	Penile (coronal sulcus), multiple chancres	Positive	1:4 (VDRL)	1.27 x 10^7^
CDC-SF006	Primary	Penile, multiple chancres	Positive	1:2 (VDRL)	4.59 x 10^3^
CDC-SF007^3^	Secondary	Perianal, ulcer	Positive	1:8 (VDRL)	4.43 x 10^4^
CDC-SF008	Primary	Penile (coronal sulcus), chancre	Positive	1:1 (VDRL)	4.50 x 10^4^

^1^ Each specimen is from a different patient.

^2^ Clinic reported non-treponemal antibody titers.

^3^ Clinical specimens that consistently propagated in rabbits.

### Serology

Fresh serum specimens from the rabbits were tested using the ASI RPR card test (Arlington Scientific, Springville, UT) and Serodia TP-PA (Fujirebio US Inc., Malvern, PA) to detect nontreponemal and treponemal antibodies, respectively. Assays were performed according to manufacturer protocols. Residual sera were stored at -80°C.

### qPCR, molecular typing and azithromycin resistance assays

DNA was extracted from remnant swab specimens from the patients’ lesion exudate using the Qiagen DNA Mini Kit as described previously [53, 54]. qPCR was performed on a Rotorgene 6000 instrument in a 50 μL final volume consisting of 20 μL extracted DNA, 25 μL of PerfeCTa qPCR Supermix (Quanta Biosciences, Beverly, MA), and 0.2 μL each of forward (TP-polA-FP 5’–CAGGATCCGGCATATGTCC– 3’) and reverse (TP-polA-RP 5’–AAGTGTGAGCGTCTCATCATTCC– 3’) primers at a final concentration of 300 nM and 0.2 μL probe (TP-polA-probe 5’–CTGTCATGCACCAGCTTCGACGTCTT– 3’) at a final concentration of 200 nM. Primers and probe were designed to be specific to the DNA polymerase I gene (*polA*) in *T*. *pallidum*. Positive (*T*. *pallidum* Nichols DNA) and no template controls were included in each run. A 10-fold serial dilution of DF-quantified *T*. *pallidum* Nichols organisms was used to construct a standard curve for quantitation of treponemes in the original patient specimens. Each rabbit isolate of *T*. *pallidum* obtained from testis extracts and tissues was tested by qPCR in triplicate. The results are stated as the mean ± standard error (SE). Molecular typing of *T*. *pallidum* isolates and azithromycin resistance marker detection were performed on the rabbit propagated isolates, as previously described, with the exception of CDC-SF008 which had a low number of spirochetes and typing was performed on the residual swab specimen[55, 56].

## Results

### Propagation of *T*. *pallidum* from cryopreserved penile ulcer specimens

The eight specimens that were used in this study were sourced from the San Francisco Municipal STD Clinic in San Francisco, CA. Clinical and CDC laboratory-derived data associated with these specimens are indicated in Table 1. Upon thawing of the cryopreserved specimens for rabbit studies at the CDC, additional qPCR and DF microscopy analyses were performed (Table 1). Examination of residual specimens by DF microscopy yielded inconclusive results due to the presence of swab material which obscured visualization. Of the eight rabbits that were initially inoculated, the two that received 1.3 ml CDC-SF011 IT or 1.3 ml CDC-SF002 IT did not develop orchitis or seroreactivity for the duration of the three-month experimental period and at necropsy (Table 2). Whole blood specimens also tested negative by PCR for both rabbits. Testes specimens at necropsy showed positive qPCR results for the rabbit inoculated with CDC-SF011, indicating an observed *T*. *pallidum* genomic equivalent of 64.29 ± 9.22 copies/ml and 0.55 ± 0.13 copies/mg in the left testis extract and tissue, respectively (Table 2). *T*. *pallidum* genomic equivalents in the right testis indicated a copy number of 192.86 ± 29.74 per ml and 1.88 ± 0.29 per mg in the extract and tissue. However, there was no evidence of *T*. *pallidum* in CDC-SF002 by PCR. Left testis extract from the CDC-SF011 inoculated rabbit, was serially blind passaged (IT) in two age-matched rabbits. However, neither rabbit yielded treponemes and although weakly positive by serology (+/-1:80 TP-PA) at the terminal end of the second passage, the rabbit remained negative for orchitis and specimens were negative by DF and PCR for both rabbits during the three-month period and at necropsy.

**Table 2:** Summary of end point measurements and *T*. *pallidum* yield where applicable for *in vivo* rabbit propagation of patient specimens.

Specimen ID	Inoculationroute^1^	Passage number^2^	Measurement				*T*. *pallidum* yield^3^ (qPCR)	DF	*T*. *pallidum* strain type^4^
			Orchitis	Serology	PCR (blood)	PCR (testes)			
CDC-SF011	IT	1	-	-	-	+	64.29/ml	-	ND^6^
	IT	2	-	+^5^	-	-	-	-	
CDC-SF002	IT	1	-	-	-	-	-	-	N/A
CDC-SF003	IV and IT	1	+	+	+	+	4.84 x 10^6^/ml	+	14*d*9f
	IT	2	+	+	+	+	1.62 x 10^6^/ml	+	
CDC-SF004	IV and IT	1	-	-	-	-	-	-	N/A
CDC-SF005	IV and IT	1	-	-	-	-	-	-	N/A
CDC-SF006	IV and IT	1	-	-	-	-	-	-	N/A
CDC-SF007	IV and IT	1	-	+	+	+	6.91 x 10^5^/ml	+	14*d*9g
	IT	2	-	+	+	+	3.11 x 10^4^/ml	+	
CDC-SF008	IV and IT	1	-	+	-	+	1.06 x 10^4^/ml	-	14*d*10c^7^
	IT	2	-	-	-	+	1.1 x 10^3^/ml	-	

^1^ IT–intratesticular, left testis; IV–intravenous.

^2^ Indicates passage number for serial passages performed.

^3^ *T*. *pallidum* yield expressed as genomic equivalents in left testis extract.

^4^ Strain typing was performed on testes samples that yielded PCR+ results.

^5^ Weakly positive TP-PA titer +/- 1:80

^6^ Isolate could not be typed due to low number of spirochetes after rabbit propagation

^7^ Strain type observed with residual lesion swab specimen in NRS

Of the remaining six specimens that were inoculated IV and IT (left testis), two specimens CDC-SF003 and CDC-SF007 successfully grew *in vivo*, while results for a third specimen, CDC-SF008, indicated infection but poor replication. None of the rabbits that were inoculated IV and IT developed disseminated skin lesions during the three-month monitoring period. The specimen CDC-SF008 resulted in seroreactivity in a rabbit 10 weeks after inoculation (TP-PA 1:5120; RPR 1:16) and was euthanized in week 12 (TP-PA 1:5120; RPR 1:64). However, whole blood yielded negative PCR results, and the animal showed negligible signs of orchitis during the entire 12-week experimental period. Processing of fresh testes tissues immediately after euthanasia and analysis by qPCR showed a *T*. *pallidum* genomic equivalent of 1.06 x 10^4^ ± 583 copies/ml and 5.71 x 10^3^ ± 1.27 x 10^3^ copies/mg in the left testis extract and tissue (Table 2), respectively. *T*. *pallidum* genomic equivalents in the right testis indicated a copy number of 288 ± 58.06 per ml and 3 ± 0.52 per mg in the extract and tissue, respectively. No treponemes were evident by DF microscopy in extracts from both testes. Serial passage in a second rabbit yielded similar results, with seronegative and PCR negative outcomes for blood during the monitoring period, and DF negative extract from the left and right testes obtained at euthanasia in week 4. However, the left testis tissue and extract yielded positive results by qPCR with a *T*. *pallidum* genomic equivalent of 1.85 x 10^2^ ± 56.25 copies/mg and 1.1 x 10^3^ ± 202 copies/ml, respectively (Table 2), with none detected in the right testis.

**Fig 2:**
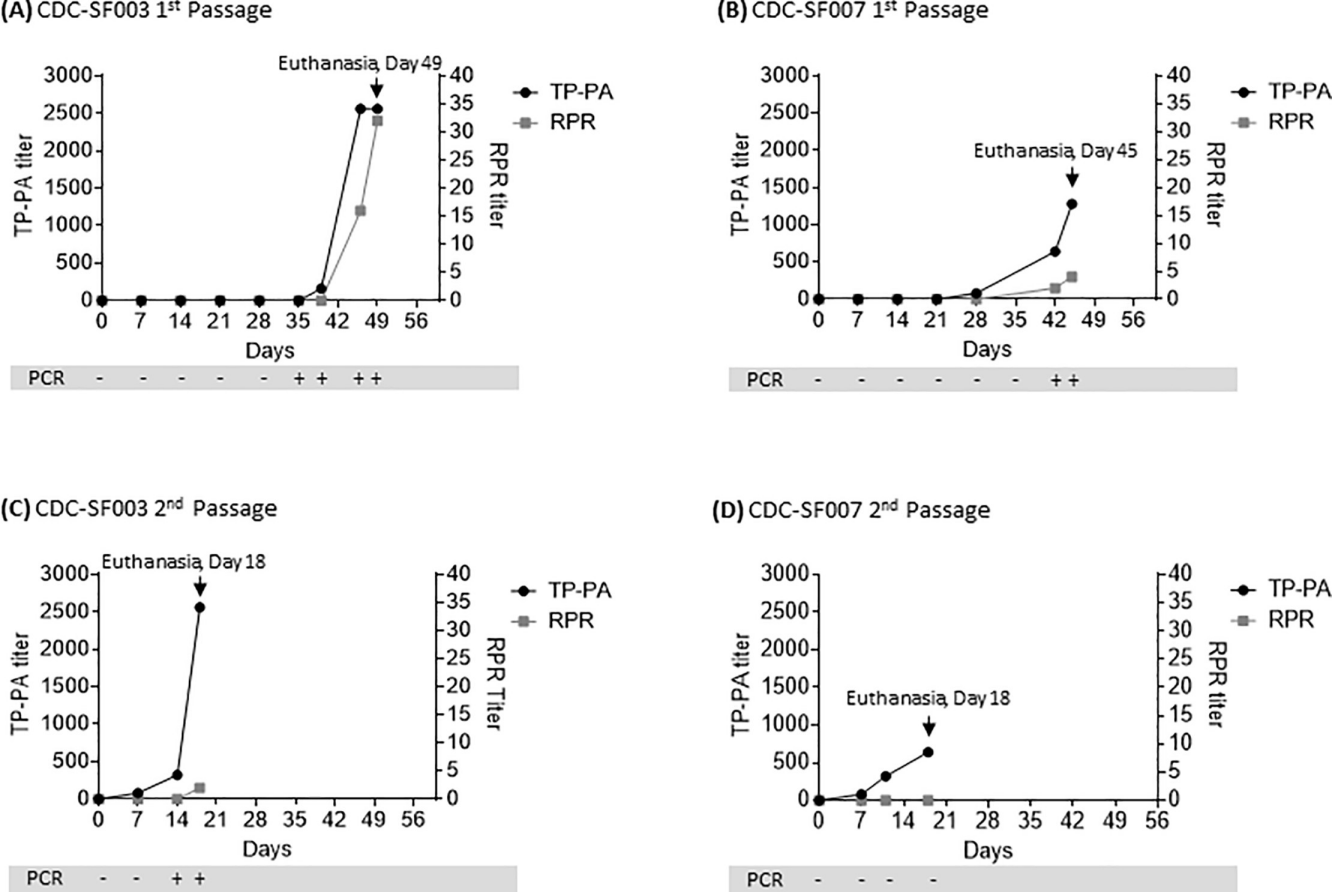
Serology results for successfully propagated specimens. Treponemal (TP-PA, black line, left axis) and nontreponemal (RPR, grey line, right axis) antibody titers in rabbits (N = 1 per passage) that were (A) inoculated IV and IT (0.6 ml inoculum per site) with the patient specimen CDC-SF003 and (B) inoculated IV and IT (0.6 ml inoculum per site) with the patient specimen CDC-SF007. Second passage was then performed for (C) CDC-SF003 inoculated IT with fresh testicular extract (1.5 ml) from the first passage rabbit and (D) CDC-SF007 inoculated IT with previously frozen and thawed testicular extract (1.5 ml) from the first passage rabbit. Whole blood was also analyzed weekly by PCR, with results shown in the grey bars for each time point.

The rabbit that was inoculated IV and IT with an equal volume (0.6 ml) of CDC-SF003 per site began to show signs of orchitis in the left testis in week 6. Serum testing indicated a weak, minimum reactive status by RPR and a treponemal antibody titer of 1:160 by TP-PA (Fig 2A). Orchitis progressed further in the following week (week 7), with antibody titers increasing to 1:16 (RPR) and 1:2560 (TP-PA). Euthanasia and necropsy were performed three days later during week 7, and the left and right testes were processed to obtain extract for further analysis. The presence and viability of treponemes in the left testis were confirmed by examination of fresh testis extract by DF microscopy, while extract from the right testis was confirmed to be DF negative. Analysis by qPCR of fresh extract yielded a genomic equivalent of 4.84 x 10^6^ ± 1.41 x 10^5^ copies/ml and 2.17 x 10^5^ ± 2.49 x 10^4^ copies/mg in the left testis extract and tissue (Table 2), respectively, while *T*. *pallidum* was also detected in the right testis extract (306 ± 32 copies/ml) and in the right testis tissue (3 ± 0.44 copies/mg). Antibody titers (Fig 2A) on the day of euthanasia were 1:32 (RPR) and 1:2560 (TP-PA). Whole blood analysis showed that the rabbit was positive for *T*. *pallidum* by PCR from week 5 and remained as such through week 7 (Fig 2A).

Similarly, for the other isolate that was successfully propagated, the rabbit that was inoculated with an equal volume (0.6 ml) of CDC-SF007 IT and IV became seroreactive in week 4 post-inoculation, with a TP-PA titer of 1:80 that increased to 1:1280 on the day of euthanasia in week 6, which is when an RPR titer of 1:4 was also observed (Fig 2B). Blood collection in week 5 was not possible due to scar tissue at the ear vein sites, and blood draw resumed the following week. Whole blood was shown to be positive by PCR in week 6, and the animal showed negligible signs of orchitis during the entire six-week experimental period. Processing of fresh testes tissues immediately after euthanasia confirmed the presence of viable treponemes in the left testis via DF microscopy, with none visible in the extract from the right testis. Analysis by qPCR showed a *T*. *pallidum* genomic equivalent of 6.91 x 10^5^ ± 6.92 x10^4^ copies/ml and 2.40 x 10^5^ ± 2.51 x 10^4^ copies/mg in the left testis extract and tissue, respectively (Table 2). No *T*. *pallidum* was detected in the right testes extracts and tissues by qPCR.

### Second passage of CDC-SF003 and CDC-SF007

To further confirm that the viability of the propagated treponemes is sustainable, live *T*. *pallidum* obtained from the left testis of the rabbit inoculated with the CDC-SF003 specimen was passaged through a second naïve, age-matched rabbit by injecting its left testis with 1.5 ml of fresh testis extract within 1 hour of the tissue processing that was performed for the first passage rabbit. The remaining fresh extract from the first passage rabbit was cryopreserved. Seroreactivity in the second passage rabbit was evident in week 1, with TP-PA titers being 1:80 while RPR was negative (Fig 2C). Orchitis was apparent in week 2 post infection, with whole blood also beginning to test positive by PCR at this time. Treponemal antibody titers (TP-PA) continued to increase to 1:320 and 1:2560 over the next two weeks, though nontreponemal titers (RPR) were minimal with a weakly reactive 1:2 titer observed in week 3 when the animal was euthanized. Testes tissue harvest and processing from the second passage rabbit were performed as described above, yielding extract from the left testis which confirmed the presence of live treponemes by DF microscopy. Analysis by qPCR showed a *T*. *pallidum* genomic equivalent of 1.62 x 10^6^ ± 2.30 x 10^4^ copies/ml and 2.32 x 10^4^ ± 695 copies/mg in the left testis extract and tissue, respectively (Table 2). As observed for the first passage rabbit, *T*. *pallidum* was also detected in the right testis extract (589 ± 54 copies/ml) and its tissue (36 ± 6 copies/mg) at the time of necropsy. Left and right testes extracts and tissues were cryopreserved as detailed above.

The second passage of CDC-SF007 also successfully yielded treponemes. The left testis extract containing live treponemes from the first passage rabbit had been cryopreserved, later thawed and inoculated into the left testis of a second rabbit. Seroreactivity was evident in week 2 (1:320 TP-PA), with treponemal titers increasing to 1:640 in week 3 when the rabbit was euthanized, though nontreponemal RPR titers remained negative (Fig 2D) and there was no indication of orchitis. Whole blood was PCR-negative while left testis tissue and extract were positive, yielding genomic equivalents of 1.23 x 10^4^ ± 2.4 x 10^3^ copies/mg and 3.11 x 10^4^ ± 2.57 x 10^3^ copies/ml, respectively (Table 2). Extract from the left testis was DF positive. The right testis was negative by both PCR and DF.

### Molecular typing and azithromycin resistance assays

Strains CDC-SF003, CDC-SF007, and CDC-SF008 were characterized as type 14*d*9f, 14*d*9g, and 14*d*10c using the 4-component molecular typing system (Table 2). All three isolates and CDC-SF011 had the A2058G mutation associated with azithromycin resistance. Due to the reduced presence of *T*. *pallidum* in the rabbit testis extract of CDC-SF011, strain typing could not be confirmed.

## Discussion

Described in this study is the successful propagation of *T*. *pallidum* from two patients’ cryopreserved syphilitic ulcer exudate using the rabbit model, with two additional patient specimens showing initial albeit unsustainable growth *in vivo*. Strain isolation in recent years has been particularly challenging, as evidenced by the limited number of published studies to date. To the best of our knowledge, while *T*. *pallidum* has been grown in rabbits using fresh blood, fresh CSF and fresh primary chancre exudate [9, 27, 31, 50] from patients diagnosed with syphilis, there have been no prior reports of adapting this method for cryopreserved lesion exudate. In addition to orchitis monitoring [9, 21, 26, 30, 57–62], FDA-cleared syphilis serology tests and a CDC-developed investigational PCR assay [63] were also performed in parallel to track infection, since these clinical specimens contain uncharacterized *T*. *pallidum* strains with unknown or unpredictable disease manifestation(s) in rabbits. Indeed, the rabbit that was injected with CDC-SF007, which successfully grew *in vivo*, did not show any discernible orchitis even when seroreactive and PCR+, while the rabbit with CDC-SF003 developed orchitis beginning in week 6 when an increase in antibody titers was first noted. Consistent with previous studies [9, 26, 61, 64, 65], these observations indicate that including additional detection methods for treponemal propagation in rabbits can avoid overlooking an active infection that could inadvertently be cleared by the immune response before treponemal harvest can be performed. Of note, when comparing serology and PCR, both methods generally showed a reactive or positive result at comparable time points post infection for a given rabbit, which aligns with a previous study [61]. Furthermore, *T*. *pallidum* was detected by PCR in whole blood, testis tissues and extracts, suggesting that a broad range of specimen types can be successfully evaluated using this molecular method.

Both rabbits that were inoculated with CDC-SF003 and CDC-SF007 developed treponemal antibodies first as shown by TP-PA when compared to the nontreponemal antibodies measured by RPR. This observation is in agreement with previous reports for both the rabbit model and patients diagnosed with syphilis, where treponemal antibodies have been shown to develop prior to nontreponemal antibodies [10–12, 66–68], albeit treponemal antibody detection in patients does not always distinguish among recent, past, and previously treated infection. The relatively rapid development of orchitis and/or seroreactivity in the second passage rabbits for CDC-SF003 and CDC-SF007 compared to the first is consistent with serial passage described in previous studies and is attributed in part to a more concentrated starting inoculum, and genetic diversification and adaptation of *T*. *pallidum* in the rabbit model [9, 27, 28].

The three strains CDC-SF003, CDC-SF007 and CDC-SF008 had different strain types (14*d*9f, 14*d*9g, and 14*d*10c) in this study suggesting that successful propagation of isolates from previously frozen specimens is unlikely to be associated with strain type; however, the sample size was small. The 14*d*9f and 14*d*9g types were reported previously in San Francisco and Vancouver, while 14*d*9f and 14*d*10c were found in Cape Town [56].

Six out of the eight cryopreserved specimens in this study failed to grow *in vivo*, with two specimens in particular (CDC-SF011, CDC-SF008) demonstrating discrepant results by serology, PCR and DF microscopy. The reason(s) for these observations is unclear and immune clearance of the treponemes in the interim between blood tests, or prior to euthanasia, cannot be ruled out. Although serology, PCR and orchitis monitoring was performed for all rabbits, it became evident that variability among individual animals and/or strains still posed a challenge for deciphering the ideal time to euthanize as not all laboratory tests and disease manifestation (orchitis) aligned. In addition, while every effort was made to maintain consistency in procedures related to specimen collection at the clinical site, there are still a number of variables to take into account. Each specimen was evaluated by DF microscopy and qPCR following rabbit inoculations at the CDC. Although the fresh lesion exudate specimens from each of the patients were confirmed to be DF positive at the clinical site, this could not be later verified for the corresponding thawed specimens at the CDC due to the presence of extraneous material from swabs used for exudate collection and cryopreservation, and the dilution of treponemes after addition of storage medium. To circumvent this issue, qPCR analysis was also performed and while a quantitative measure of *T*. *pallidum* in residual inoculum was obtained, viability cannot be determined by this method. Given that an appreciable loss in viability occurs during any freeze-thaw cycle(s), it is unknown whether this impacted growth *in vivo*. Ideally, simultaneous comparison of propagating a fresh sample and a thawed, previously frozen sample for a given patient specimen would help address the effect(s) of freeze-thaw on viability of lesion exudate specimens though this would again require proximity between the clinical site and animal facility. Residual inocula containing CDC-SF003 and CDC-SF007 that were successfully propagated each showed a treponemal concentration that is in the median range among the eight specimens that were tested by qPCR. Thus, there does not appear to be any correlation between the concentration of *T*. *pallidum* in the clinical specimen and likelihood of propagation in rabbits. The seroreactive status of the patients from whom the specimens were collected was also considered as a potential factor affecting propagation in rabbits. Patients from whom CDC-SF003 and CDC-SF007 were collected showed VDRL antibody titers of 1:4 and 1:8, respectively, which are similar if not lower than the titers for the other patients whose specimens did not propagate successfully in the rabbits. Additionally, the reported antibody titers for some of the patients were derived from either VDRL or RPR testing and being different assays, these results are not interchangeable. Thus, it is not possible to discern a trend based on patient serostatus alone. However, as more specimens are tested for rabbit propagation, a larger sample size may shed light on specimen characteristics, if any, that favor or lower the probability of *in vivo* growth, and these studies are ongoing.

Another experimental variable to consider in this study is the route of inoculation. Since IT inoculation did not yield treponemes in testes extracts for the CDC-SF011 and CDC-SF002 strains in initial experiments, inoculations for the remaining clinical specimens were divided between the IT and IV route for each rabbit, to facilitate systemic infection and formation of disseminated skin lesions that could potentially be used as an alternative source of treponemes for subsequent passage, in the event that growth in the testis was not sufficient or successful. However, no lesions developed over the three month monitoring period in any of the rabbits that were inoculated IV and IT. A caveat is that the dual inoculation route approach used in this study essentially halved the dose for each site, potentially reducing the chances of lesion development and/or sustained treponemal growth in the testes. However, this method proved successful for IT growth of CDC-SF003 and CDC-SF007 that had inoculum genomic equivalents on the order of 10^4^/ml in the original specimens, which is less than or equivalent to that of the other specimens that did not grow in the testes but were also subjected to the same inoculation method. These results further highlight the complexity of the variables involved, particularly that *in vivo* propagation of clinical isolates may not depend on treponemal count alone. Other factors that are independent of inoculum concentration and route, and instead intrinsic to the strain’s adaptability and/or viability cannot be ruled out. Of note, the right testis of both the first and second passage CDC-SF003 rabbits showed low but detectable levels of *T*. *pallidum* by qPCR even though it was not the site in which inoculum was injected, which is an observation that has been previously reported [69]. However, *T*. *pallidum* was not observed in the right testis or whole blood of the second passage rabbit inoculated with CDC-SF007 and may be due to the overall lower number of spirochetes compared to the second passage of CDC-SF003.

In summary, the method described herein shows that propagation of *T*. *pallidum* from patients’ cryopreserved lesion exudate specimens is reproducible and feasible, broadening the scope of specimen types that can be used in the rabbit model. Performing serology and PCR techniques in parallel with orchitis monitoring can also guide propagation timelines for uncharacterized clinical strains in rabbits. High yields of viable *T*. *pallidum* CDC-SF003 and CDC-SF007 on the order of 10^5^−10^6^ treponemes/ml were obtained from up to two passages, producing sufficient stock for cryopreservation and facilitating future studies. While further method optimization is necessary and ongoing, our findings provide an additional path for isolation and sustained growth of clinical *T*. *pallidum* strains, which could also be potentially applied to yaws and bejel isolates. This approach could facilitate complementary whole genome sequencing and pathology studies to decipher strain-specific phenotypes, potentially helping identify patients at risk for syphilis complications such as neurosyphilis and ocular syphilis. Additional research such as diagnostic assay development and vaccine studies based on antigen expression from current strains, which require an appreciable amount of treponemal material, may also benefit from our findings.
